# Positron Emission Tomography in Merkel Cell Carcinoma

**DOI:** 10.3390/cancers12102897

**Published:** 2020-10-09

**Authors:** Christos Sachpekidis, Polytimi Sidiropoulou, Jessica C. Hassel, Nikolaos Drakoulis, Antonia Dimitrakopoulou-Strauss

**Affiliations:** 1 German Cancer Research Center, Clinical Cooperation Unit Nuclear Medicine, 69120 Heidelberg, Germany; a.dimitrakopoulou-strauss@dkfz.de; 2 1st Department of Dermatology-Venereology, Faculty of Medicine, National and Kapodistrian University of Athens, “A. Sygros” Hospital for Cutaneous & Venereal Diseases, GR-16121 Athens, Greece; sidiropouloupolytimi@gmail.com; 3 Department of Dermatology and National Center for Tumor Diseases, University Hospital Heidelberg, 69120 Heidelberg, Germany; Jessica.Hassel@med.uni-heidelberg.de; 4 Research Group of Clinical Pharmacology and Pharmacogenomics, Faculty of Pharmacy, School of Health Sciences, National and Kapodistrian University of Athens, GR-15771 Athens, Greece; drakoulis@pharm.uoa.gr

**Keywords:** Merkel cell carcinoma (MCC), positron emission tomography/computed tomography (PET/CT), ^18^F-fluorodeoxyglucose (^18^F-FDG), ^68^Ga-labeled somatostatin analogues

## Abstract

**Simple Summary:**

There is currently no consensus on a widely accepted algorithm for imaging Merkel cell carcinoma (MCC) patients. Baseline, tomographic imaging is not generally recommended in early-stage disease, but its value in locally advanced and/or distant metastatic MCC has been well established. In this context, the hybrid imaging modality positron emission tomography/computed tomography (PET/CT) is increasingly applied in the workup of metastatic or unresectable MCC, providing essential information for staging, restaging, and treatment monitoring of the disease. Although the role of PET/CT in the management of loco-regional MCC is still limited and less well-defined, current evidence suggests its important contribution also in cases of localized MCC. Herein, we provide a structured literature review summarizing the most important studies on the role of PET or PET/CT with different radiopharmaceuticals in the clinical care of MCC.

**Abstract:**

Merkel cell carcinoma (MCC) is a rare neuroendocrine skin malignancy usually arising as a nonspecific nodule on sun-exposed areas of the head and neck. Given the poor prognosis of this aggressive tumor, assessment of disease burden in pre- and post-treatment care may ensure an optimal management with significant implications for patient surveillance and prognosis. Although imaging has established its role in locally advanced or distant metastatic MCC, a standard imaging algorithm is yet to be determined and respective recommendations are mainly based on melanoma. Positron emission tomography/computed tomography (PET/CT) is increasingly evolving as a valuable imaging tool in metastatic or unresectable MCC, mostly utilizing the glucose analogue ^18^F-fluorodeoxyglucose (^18^F-FDG) as a radiotracer. Despite being inferior in detecting the disease in its early stages compared to the “gold standard” of sentinel lymph node biopsy, recent evidence suggests an important role for ^18^F-FDG PET/CT in the routine workup of localized MCC. Moreover, ^68^Ga-labeled somatostatin analogues have been employed as PET tracers in the field of MCC with promising, yet comparable to ^18^F-FDG, results. This article provides a structured literature review of the most important studies investigating the role of PET or PET/CT in the clinical practice of MCC.

## 1. Introduction

Merkel cell carcinoma (MCC) is a rare neuroendocrine skin malignancy usually arising as a red/bluish or flesh-colored nodule on sun-exposed areas of the head or neck in elderly, fair-skinned, and/or immunocompromised patients [1,2]. Due to its aggressive nature and nonspecific clinical features, diagnostic delays correlated with high rates of regional (26%) or distant (8%) metastatic spread at presentation are common [1]. Following resection of the primary lesion, recurrence will occur in nearly 30% of cases [3]. The five-year overall survival (OS) is reported to be as high as 51%, 35%, and 14% for local, nodal, and distant disease, respectively [1,4]. Based on the poor prognosis of MCC, assessment of disease burden in pre- and post-treatment care may thus ensure an optimal management adding diagnostic value in staging/restaging and providing therapeutic guidance.

For the management of MCC, the National Comprehensive Cancer Network (NCCN) and the European consensus-based interdisciplinary guidelines propose specific treatment algorithms based on disease extent at presentation [5]. Initial management of loco-regional MCC typically involves complete surgical excision of the tumor lesion (wide, local excision with resection margins of 1–2 cm or Mohs micrographic surgery) followed by adjuvant radiotherapy of the primary site. In the metastatic setting, a case-by-case multidisciplinary approach is recommended. While mono- or poly-chemotherapy alone or in combination with radiotherapy has commonly been used to treat advanced forms, innovative therapies, mostly utilizing immune checkpoint inhibitors of the programmed death-ligand 1 (PD-L1; avelumab) and programmed cell death-1 (PD-1; pembrolizumab, nivolumab) axis, should be considered, if indicated. Moreover, targeted molecular therapies, i.e., somatostatin (SST) analogues, tyrosine kinase inhibitors, and mammalian target of rapamycin inhibitors, are currently in development [6,7,8].

Among imaging modalities, brain magnetic resonance imaging (MRI), neck/chest/abdomen/pelvis computed tomography (CT), and/or whole-body positron emission tomography/computed tomography (PET/CT) appear to be essential for evaluating MCC, especially in advanced stages. However, a standard imaging protocol remains to be established [1,2,9]. Given the much lower incidence compared to melanoma, current MCC imaging guidance reflects the melanoma guidelines [5]. In line with melanoma, whole-body baseline imaging is not generally recommended in early-stage MCC but is of value in locally advanced or distant metastatic disease [5]. However, emerging evidence suggests that baseline imaging enables detection of clinically occult metastatic spread in 12.5% of “localized” MCC cases [10].

In this setting, PET/CT imaging, combining the functional information of PET with the anatomic details of CT, offers superior diagnostic capabilities in several malignancies including MCC [11,12,13]. Particularly, PET/CT using the radiotracer ^18^F-fluorodeoxyglucose (^18^F-FDG) is evolving as a powerful imaging tool in the management of MCC, providing high levels of sensitivity and specificity in documenting the disease burden [13]. Herein, we provide a structured literature review summarizing the most important studies on the role of PET or PET/CT in the clinical practice of MCC.

## 2. Search Strategy and Study Selection

The PubMed/MEDLINE and Scopus databases were searched (last updated in August 2020) for studies investigating the performance of PET and/or PET/CT in MCC patients. The search algorithm was based on the following keywords: “Merkel cell carcinoma”, “MCC”, “Positron Emission Tomography”, “Positron Emission Tomography/Computed Tomography”, “PET”, “PET/CT”, “PET-CT” AND imaging, as well as on combinations of these terms. Additional relevant references were also isolated from citations in the reviewed articles. We focused only on non-preclinical data from English-language medical literature. Of the 22 original articles selected, the full-text versions were retrieved and discussed in this review (Table 1).

## 3. ^18^F-FDG PET/CT in MCC

^18^F-FDG, a glucose analogue radiolabeled with fluorine-18 (^18^F), is the major workhorse in PET imaging. The rationale for using this radiotracer in nuclear oncology is based on the increased glucose uptake encountered in tumor cells. ^18^F-FDG is actively transported and phosphorylated into cancer cells but, unlike glucose, it cannot undergo further metabolism and remains intracellularly trapped, thus enabling PET/CT to detect areas of disease activity and spread by illustrating functional changes between normal and malignant tissue [36,37].

Currently, ^18^F-FDG PET/CT has emerged at the forefront for various oncologic applications, including melanoma, lymphoma, lung, head/neck, and colorectal cancer [38,39,40,41,42]. Apart from providing both functional and anatomical information in a single session, this modality also enables quantification of tumor radiotracer uptake via the standardized uptake value (SUV), an index reflecting the intensity of tracer activity in the visualized part of interest [43]. This allows an objective characterization of PET/CT scans beyond the ‘standard’ visual evaluation of ^18^F-FDG uptake with significant implications on patient follow-up and prognosis [44,45,46,47].

MCC tumors are typically highly metabolic, showing intense ^18^F-FDG uptake at PET [13,48]. Particularly for primary lesions, increased SUV max values (4.0–6.5) have been reported [48]. Thus, ^18^F-FDG appears to be an efficient PET tracer in this setting. In the following section, the most important studies as well as significant milestones regarding the use of ^18^F-FDG PET and PET/CT in MCC will be presented.

### 3.1. Initial Studies

The first reported use of ^18^F-FDG PET in the field of MCC involved a female patient with recurrent disease in 1998. Since baseline scans revealing multiple ^18^F-FDG-avid lesions were suggestive of metastases, isolated limb chemotherapy (melphalan plus tumor necrosis factor) was introduced. Post-treatment PET images showed complete metabolic response (CMR) of the lesions, demonstrating for the first time the potential utility of ^18^F-FDG PET for MCC staging/restaging, and monitoring therapy response [49]. Since then, an era of PET emerged, in which several case articles supported the favorable performance of ^18^F-FDG PET in the clinical care of MCC [14,15,50,51,52,53,54].

In 2006, Belhocine and colleagues were the first to retrospectively study the diagnostic accuracy of ^18^F-FDG PET or PET/CT in a case series of 11 MCC patients, comparing the findings with histological or clinical and radiological (CT, MRI, and bone scan) follow-up data. ^18^F-FDG PET was proven contributive in 10/11 cases, while it revealed second unexpected neoplasms in 4/11 patients. Overall, sensitivity and specificity of the modality in detecting MCC and other malignancies were 92% (11 true positive (TP), 1 false negative (FN)) and 100% (3 true negative (TN), 0 false positive (FP)), respectively [17]. Similar results were obtained in the same year in a case series of six MCC patients, where PET/CT (12 examinations) was TP in nine, TN in seven, FP in one, and FN in one lesion [18]. These early, promising data paved the way for clinical research on larger MCC patient cohorts, which will be discussed below.

### 3.2. Regional Lymph Node Evaluation

Since the sentinel lymph node (SLN) status is a major predictor of overall and disease-free survival, SLN biopsy (SLNB) should be regarded as essential or standard of care for MCC patients, comprising the most reliable staging tool for identifying subclinical nodal disease [5,55].

A number of studies have compared the information obtained by ^18^F-FDG PET or PET/CT with the “gold standard” of pathological/biopsy nodal evaluation, reporting quite different levels of sensitivity [23,24,33]. In the retrospective study by Colgan et al. [23], the findings of different imaging approaches (CT, MRI, PET, and PET/CT) were compared to conventional histology after SLNB and/or elective lymph node dissection (LND). Regarding detection of nodal spread, CT, MRI, and ^18^F-FDG PET or PET/CT exhibited a sensitivity of 47%, 0%, and 83%, and a specificity of 97%, 86%, and 95%, respectively. However, in the study by Hawryluk et al. [24], PET/CT was reported to be less capable of detecting loco-regional disease (3/21 scans; 14%); among PET/CT “negative” results (18/21 scans; 86%), 13 (72%) cases of micrometastatic disease were revealed only by means of immunohistochemistry. Another retrospective analysis of 16 stage I-II MCC patients recorded a concordance between PET/CT and histopathological findings in one of 10 patients with histologically positive nodes [33]. As similar findings have been reported for other imaging modalities [56], the NCCN panel does not recommend routine baseline imaging for clinically node-negative patients with localized MCC [5].

However, Singh et al. [10], in a recent large-scale study (*n* = 584), supported that baseline cross-sectional imaging is frequently positive, detecting occult metastatic disease in a non-negligible number of MCC cases. In this cohort, imaging upstaged 13.2% (65/492) of patients without clinically evident regional spread (8.9% in regional nodes, 4.3% in distant sites), markedly affecting management and prognosis. These findings are of high clinical importance and suggest that the current melanoma-derived imaging recommendations may be of questionable value in MCC management, underlying the potential role of imaging in routine screening of MCC patients with clinically uninvolved regional nodes.

### 3.3. Distant Metastasis Staging/Impact on Management

Compared to loco-regional MCC, the role of ^18^F-FDG PET/CT in assessing the burden of metastatic disease appears to be essential and, certainly, more well-defined. Indeed, several studies have sought to evaluate the impact of ^18^F-FDG PET/CT on stratification and management of MCC patients at virtually any stage of the disease with a reported sensitivity and specificity ranging between 86–100% and 89–100%, respectively. Notably, almost all studies have demonstrated that PET/CT, as part of the initial diagnostic workup, resulted in restaging, guiding therapeutic plans in 6–46% of cases [19,20,21,24,26,27,28,30,32,34] (Table 1).

In a first attempt to evaluate the effect of different morphological and functional imaging approaches on tumor staging and post-treatment evaluation, Peloschek et al. [20] screened 16 MCC patients with sonography, CT, MRI, and PET, comparing the findings with a combined standard of reference (histopathology, clinical, and/or radiological follow-up). Overall, ^18^F-FDG PET had a sensitivity of 85.7% and specificity of 96.2%, while the combined sensitivity and specificity for morphological imaging methods was 95.5% and 89.1%, respectively. Moreover, in regions where PET and conventional imaging correlated to the standard of reference, ^18^F-FDG PET showed 85% sensitivity and 95% specificity, while conventional imaging yielded 95% and 90%, respectively. No significant differences between the methods tested were observed.

In a retrospective chart review comprising 18 MCC cases, most staged as II/III disease, Concannon et al. [19] reported that ^18^F-FDG PET/CT resulted in restaging and influenced management plans in seven (33%) and nine (43%) cases, respectively. The modality showed a 94% sensitivity for histologically proven disease (or 100% of all lesions >5 mm). Accordingly, in the series by Maury et al. [21] (*n* = 15), ^18^F-FDG PET/CT performed at initial staging and/or during follow-up led to significant changes in disease status and management in 46% of cases compared with clinical examination alone. The sensitivity, specificity, positive predictive value (PPV), and negative predictive value (NPV) were, respectively, 89%, 100%, 100%, and 93% for both CT and PET/CT.

In the study by Hawryluk et al. [24], PET/CT conducted as part of the baseline workup upstaged 16% of patients, mainly in the more advanced stages. Regarding surveillance, their study confirmed the increased efficacy of the modality in detecting sites of metastatic disease missed on CT, especially in bone/bone marrow.

In line with previous reports, changes in tumor status as a result of PET/CT (39 scans) occurred in 20% (4/20) of cases, altering treatment decision making in 15% (3/20) [27]. Similarly, Ben-Haim et al. [32], in a cohort of 46 MCC patients, also reported changes in disease stage and management in 26% and 15% of cases, respectively. In another study of 23 MCC cases explored with ^18^F-FDG PET/CT (66 scans) at initial diagnosis or during subsequent monitoring, the modality exhibited 97% sensitivity, 89% specificity, 94% PPV, and 94% NPV. At initial presentation, PET/CT was able to restage tumor status in 39% of patients, modifying therapeutic plans in 33% of cases [28].

Siva et al. [26] retrospectively reviewed the clinical impact of PET imaging on the staging and management of 102 MCC patients. PET staging results had an impact on management in 37% of patients (*p* < 0.003) and differed from conventional staging in 22% of cases. In stratification by PET-defined stage, the five-year OS was 67% in stage I/II patients but only 31% in stage III patients (log-rank *p* < 0.001). On multivariate analysis, PET stage was significantly associated with OS (*p* < 0.001). This MCC cohort was partly followed up and evaluated a few years later. After definitive treatment of 62 patients, the impact of follow-up ^18^F-FDG PET on disease restaging, including identifying patients suitable for salvage treatment, was high in 45%, medium in 11%, and low in 43% of cases. With regard to prognosis, the status of post-treatment PET was reported to be highly prognostic of the OS, as patients who achieved a CMR assessed via PET had a two- and five- year OS of 88% and 68%, respectively, compared to 15% one-year OS in cases with residual activity [30].

In 2017, the first prospective phase II study, involving 58 MCC patients with IIA-IIIB-stage disease, evaluated the role of ^18^F-FDG PET in the management of MCC (Trans Tasman Radiation Oncology Group TROG 09.03 trial). Pre-treatment scans showed a sensitivity of 94.7%, a specificity of 88.2%, a PPV of 94.7%, and a NPV of 88.2%. Initial PET screening also provided treatment guidance in 27.6% of cases; upstaging occurred in 25.9% with no instances of downstaging. Contrary to Byrne et al. [30], no prognostic impact related to post-treatment PET was detected [34].

In the largest-to-date cohort of 352 MCC patients explored with PET/CT, Singh et al. [10] recently investigated the clinical utility of baseline cross-sectional imaging (CT, PET/CT, or MRI) focusing on patients presenting with primary cutaneous MCC and no evident distant metastatic spread. As mentioned above, imaging upstaged one in eight cases with no clinically evident regional spread, while 10.8% (10/92) of clinically node-positive patients were upstaged to distant metastatic disease. Of note, in this cohort PET/CT was more precise in accurate disease staging than CT, upstaging 16.8% of 352 cases compared to 6.9% of 231 cases who underwent CT alone (*p* = 0.0006) [10]. Table 2 provides a summary of the published studies involving a direct comparison between ^18^F-FDG PET or ^18^F-FDG PET/CT and CT.

In the era of immunotherapy, the advent of immune checkpoint inhibitors has profoundly enriched the treatment landscape of several malignancies, including melanoma, offering crucial survival benefits. Although there are no randomized comparative trials demonstrating the superiority of immune checkpoint blockade over conventional chemotherapy in metastatic MCC, preliminary results are rapidly becoming promising for PD-L1/PD-1 inhibitors [57,58]; avelumab, nivolumab, and pembrolizumab are currently recommended as first-line, systemic treatment options for advanced MCC [5].

**Figure 1:**
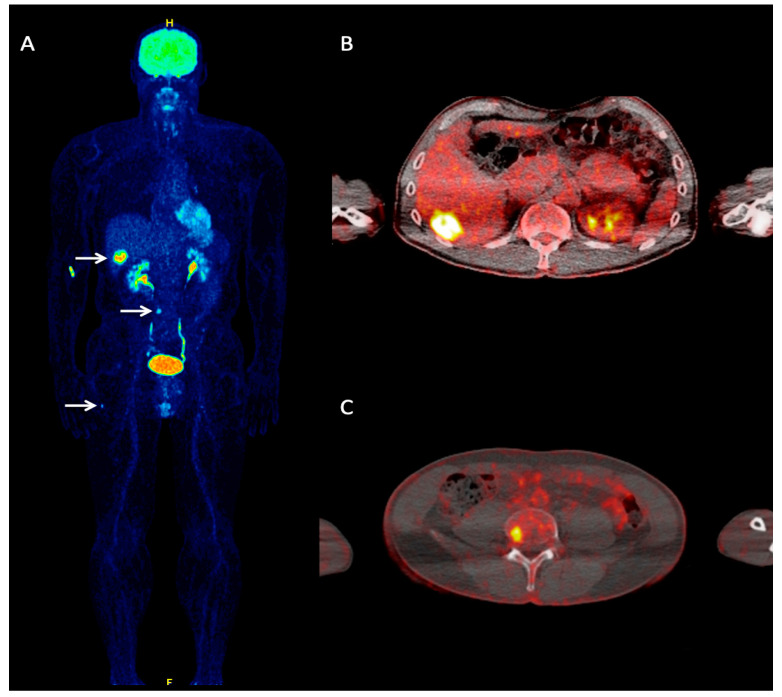
A 60-year-old Merkel cell carcinoma (MCC) patient referred to our department for staging purposes due to clinical suspicion of hepatic metastases. Whole-body ^18^F-FDG PET maximum intensity projection (MIP) (**A**) demonstrated foci of increased tracer uptake in the liver, lumbar spine, and right femur, corresponding to metastases (arrows). Transaxial, fused ^18^F-FDG PET/CT at the hepatic level (**B**) shows a focal site of increased tracer uptake in liver segment VI, corresponding to a hepatic metastasis. Transaxial, fused ^18^F-FDG PET/CT of the lower abdomen (**C**) shows pathologic tracer accumulation in the fourth lumbar vertebrae, representing an osseous metastasis.

**Figure 2:**
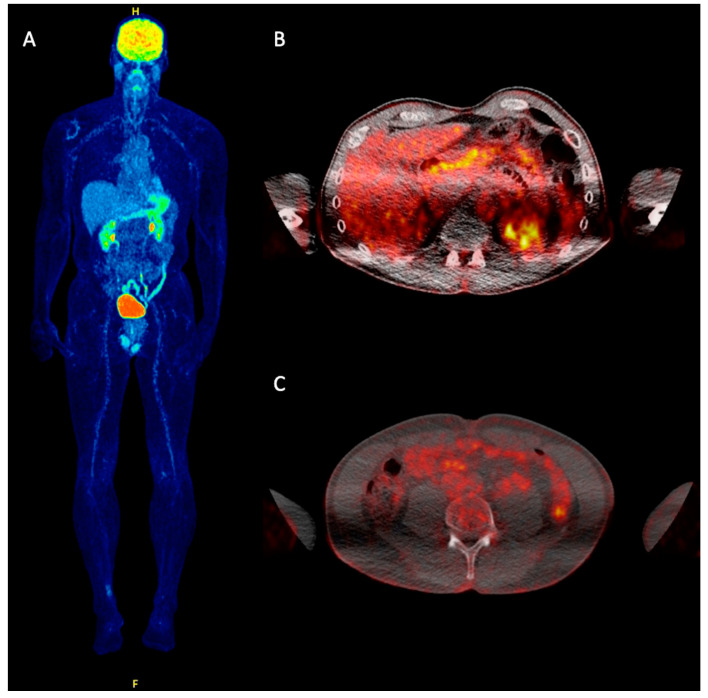
Post-treatment follow-up ^18^F-FDG PET/CT of the same patient as in Figure 1 after one year. Following anti-programmed death-ligand 1 (PD-L1) immunotherapy (avelumab), both whole-body MIP (**A**) and transaxial, fused ^18^F-FDG PET/CT images (**B**,**C**) demonstrated complete metabolic remission (CMR) of the previously observed metastases as response to treatment.

**Figure 3:**
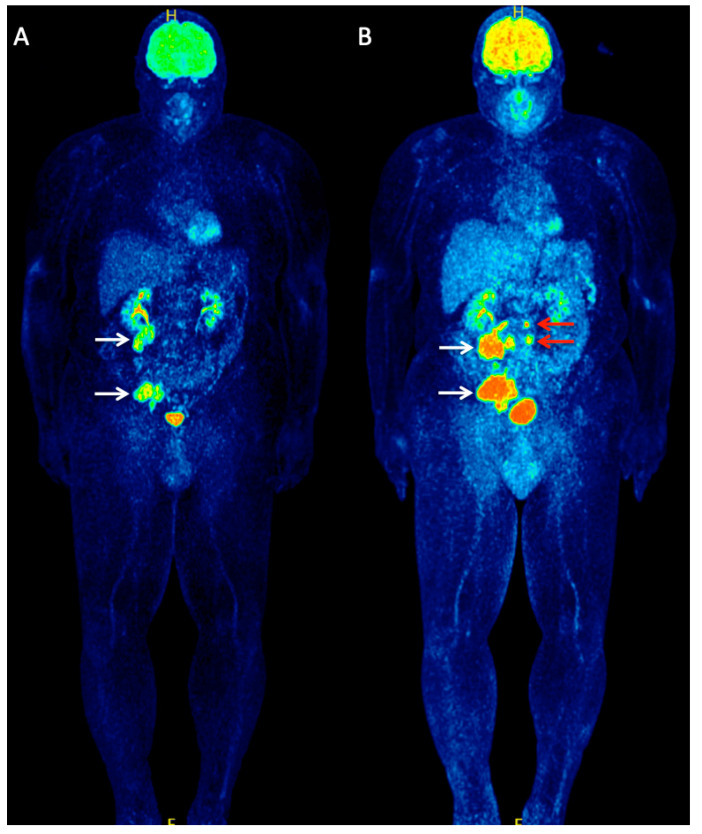
A 56-year-old patient with metastatic MCC of unknown primary referred to our department for staging purposes before initiation of immunotherapy. Whole-body ^18^F-FDG PET (MIP) (**A**) demonstrated two large hypermetabolic lesions in the abdomen and pelvis, corresponding to lymph node metastases (white arrows). The patient received treatment with the cytotoxic T-lymphocyte-associated protein 4 (CTLA-4) inhibitor, ipilimumab, considered at the time of scanning as a potentially beneficial immunotherapeutic agent for MCC. After receiving two cycles of ipilimumab, the patient underwent an interim follow-up ^18^F-FDG PET/CT for early treatment response evaluation. Whole-body ^18^F-FDG PET (MIP) (**B**) revealed a clear disease progression with an increase in size and metabolism of the previously observed metastases (white arrows) but also detected newly appeared metastatic retroperitoneal lymph nodes (red arrows).

In this evolving field, treatment response evaluation is now at the forefront of cancer management, given the different mode of action between immunotherapy and conventional chemotherapy [59,60,61]. Although ^18^F-FDG PET/CT has yielded favorable results for response assessment in melanoma immunotherapy [62,63], there is still little evidence to unravel its full potential for evaluating immunotherapy response in the field of MCC, mainly due to the rarity of this tumor. However, considering the melanoma-derived data as well as published MCC case reports/series and summarizing the experience from our institution, the modality is emerging as an attractive tool for monitoring therapeutic outcomes, enabling the evaluation of tumor burden at different time points over the course of treatment [64,65,66,67,68] (Figure 1, Figure 2 and Figure 3).

### 3.4. Limitations of ^18^F-FDG PET/CT

Disadvantages of ^18^F-FDG PET/CT include its limited availability mainly because of its high cost compared to conventional imaging modalities. Since ^18^F-FDG uptake is not specific for cancer, both false positive (i.e., inflammation, post-surgical areas, recent chemotherapy, fractures) and false negative (i.e., hyperglycemia, recent high-dose steroid therapy) results may be encountered. Moreover, the application of PET/CT in specific body areas, such as the brain, heart, and kidneys, may occasionally be suboptimal due to increased physiologic radiotracer uptake in these organs [43].

## 4. Non-^18^F-FDG PET Tracers in MCC

This section intends to address the most important non-^18^F-FDG PET tracers that have been utilized as potential imaging biomarkers in the field of MCC.

### 4.1. Somatostatin (SST) Analogues’ Imaging

Imaging with radiolabeled SST analogues has been well established in the management of neuroendocrine tumors (NETs) [69]. For high-grade NETs, like MCC, SST receptor (SSTR) expression can also be utilized for visualization of disease burden and potentially theranostics [48]. In this setting, ^111^In-pentetreotide scintigraphy (OctreoScan) has been used in the workup of MCC, with reported results ranging from initially promising to reserved or even disappointing [70,71,72]. In the largest cohort study, 85% of MCC patients (*n* = 39) had at least some degree of ^111^In-pentetreotide uptake in SSTR scintigraphy, with the majority (75%) showing low to medium tracer uptake. However, the SSTR expression status, assessed by scintigraphy, did not significantly correlate with clinical outcomes of SSTR-targeted therapy [73].

Although extensive comparative studies of OctreoScan and ^18^F-FDG PET have not yet been carried out, a limited number of case reports and case series studies have all demonstrated the superiority of PET, which can partly be due to its improved image resolution and sensitivity over scintigraphy [16,22,50]. In particular, regarding disease staging, Lu et al. confirmed the enhanced performance of ^18^F-FDG PET/CT compared to scintigraphy in a series of nine MCC cases, upstaging 56% of patients and altering clinical decisions in all cases. Interestingly, no lesions identified by OctreoScan were missed on PET/CT [22].

The introduction of ^68^Ga-labelled dodecane tetraacetic acid (DOTA)-peptides with high affinity for SSTR in PET imaging, such as ^68^Ga-DOTA-d-Phe^1^-Tyr^3^–Octreotide (DOTATOC), ^68^Ga-DOTA-Tyr^3^-Octreotate (DOTATATE), and ^68^Ga-DOTA-Nal3-Octreotide (DOTANOC), has optimized NETs’ detection and characterization, providing higher diagnostic efficiency and accuracy over scintigraphic approaches [74,75,76].

Concerning MCC, ^68^Ga-labeled SST analogues’ PET and PET/CT have shown highly promising results. Salavati and coauthors in 2012 were the first to perform PET/CT examinations using ^68^Ga-DOTATOC and ^18^F-FDG in a patient with stage IV MCC, demonstrating similar performance of both tracers in detecting metastatic disease. Based on the intense uptake of SST analogues by metastatic lesions, the authors took a step further and administered adjuvant peptide receptor radionuclide therapy (PRRNT) with ^177^Lu-DOTATATE in combination with doxorubicin chemotherapy, implementing the first documented ‘theranostic’ strategy in MCC management. However, follow-up PET/CT scans showed mixed patterns of response; despite a significant decline in tumor size and SSTR expression at specific sites, progressive disease with new skin and nodal lesions was also detected [77].

A similar approach was subsequently adopted by Schmidt et al. [25] in two MCC cases with extensive lymph node involvement. Following confirmation of SSTR expression in metastatic lesions using ^68^Ga-DOTATATE PET/CT, both patients were introduced to combination therapy with PRRNT (^90^Y-DOTATATE or ^177^Lu-DOTATATE) and capecitabine followed by external beam radiotherapy in one case. Despite a temporary partial response in both patients, however, fatal outcomes could not be prevented [25].

Further case reports have supported the usefulness of ^68^Ga-labelled tracers for MCC detection and management in the context of theranostics [78,79,80]. In 2014, Buder et al. [29] retrospectively studied the largest MCC cohort (*n* = 24) using SSTR-PET with ^68^Ga-DOTATOC and ^68^Ga-DOTATATE radiotracers in comparison to CT. Nodal, bone, and soft-tissue metastases were revealed by SSTR-PET with a sensitivity of 73%, 100%, and 67%, respectively. Based on PET findings, four (17%) patients were upstaged and management was modified in three (13%) cases.

One year later, another retrospective analysis comprising 23 MCC patients evaluated the role of ^68^Ga-DOTA-peptides PET/CT. Overall, the modality showed a sensitivity, specificity, and diagnostic accuracy of 92%, 73%, and 83%, respectively. Higher diagnostic accuracy was obtained for staging compared to restaging (88% vs. 73%; *p* = 0.7) and for ^68^Ga-DOTANOC compared to ^68^Ga-DOTATOC or ^68^Ga-DOTATATE (100% vs. 71% and 75%, respectively; *p* = 0.56). Disease management was influenced by PET/CT findings in almost 30% (7/23) of cases [31].

Further, Taralli et al. [35] compared the impact of ^18^F-FDG and ^68^Ga-labeled SST-analogues’ PET/CT on staging, restaging, or treatment response evaluation in a series of 15 MCC patients. Using histology or clinical/radiological follow-up as the reference standard, both approaches showed good and comparable diagnostic performance; ^18^F-FDG and ^68^Ga-labeled SST-analogues’ PET/CT had both a sensitivity of 100% and a specificity of 85.7% and 71.4%, respectively, with no significant differences. The authors thus concluded that ^68^Ga-SST analogue PET/CT cannot replace but rather supplement ^18^F-FDG PET/CT, according to clinical indication.

### 4.2. ^18^F-Fluorodihydroxyphenylalanine (^18^F-DOPA)

6-Fluoro-(^18^F)-L-3,4-dihydroxyphenylalanine (^18^F-DOPA) is a neutral amino acid analogue employed as a PET tracer. When injected intravenously, this molecule can cross the blood–brain barrier to reach the dopaminergic neurons where it is used as a precursor of the neurotransmitter dopamine. In the field of nuclear oncology, the main clinical application of imaging with ^18^F-DOPA is for the management of NETs and brain tumors [81,82]. On the basis of the knowledge that the amino acid DOPA is a precursor of melanin, ^18^F-FDOPA PET has also been experimentally applied in melanoma patients in combination with ^18^F-FDG. However, its sensitivity as well as the tracer uptake in melanoma lesions was lower in comparison to ^18^F-FDG [83].

Given that NET cells are able to uptake, decarboxylate, and store biogenic amines [16], ^18^F-DOPA PET has been used in few case series of MCC patients. In their 2006 retrospective case study (*n* = 3), Talbot et al. [16] were the first to evaluate and compare the performance of ^18^F-DOPA PET, ^18^F-FDG PET, and SSTR scintigraphy. Despite ^18^F-FDOPA uptake by MCC lesions, ^18^F-FDOPA PET added no further information than ^18^F-FDG PET in the two TP patients, while it provided inferior contrast of images. Moreover, in one case suspected of recurrence at scintigraphy and with inconclusive ^18^F-FDG PET, the ^18^F-FDOPA PET result was proven TN [16].

A few years later, Peloschek et al. [20], in a retrospective chart review, compared imaging findings obtained via ^18^F-DOPA PET with a standard of reference (histopathology or clinical/radiological follow-up) in five MCC cases. FDOPA results were negative in all anatomical sites (19 TN, two FN), correlating to the standard of reference in 21/144 regions. These findings further suggested the limited clinical utility of ^18^F-FDOPA PET in MCC diagnostics.

Given the limited, but discouraging, data on the use of ^18^F-DOPA PET in MCC, as well as the practical and logistical issues regarding the complicated labeling process of the tracer, the potential role of this modality in the management of MCC seems to be rather poor.

## 5. Conclusions

In summary, there is currently no consensus on a widely accepted imaging algorithm for MCC, and respective recommendations are still based mainly on melanoma. While baseline imaging is not generally encouraged in early-stage MCC, its value in locally advanced and/or distant metastatic disease has been well established. In this context, PET/CT, mostly utilizing ^18^F-FDG, is increasingly applied in the workup of metastatic or unresectable MCC, providing essential information for initial staging, therapy response evaluation, and monitoring of recurrent disease. Although the accuracy of the modality in detecting the disease in its early (I/II) stages appears to be inferior compared to SLNB, current evidence suggests an important contribution also in cases of localized MCC. In addition, ^68^Ga-labeled SST analogues have also been used as PET tracers with promising results, given that SSTR expression may be utilized as a potential target for visualizing MCC, according to clinical indication. Despite the limited experience, ^18^F-DOPA PET imaging seems to be less valuable in the field of MCC diagnostics.

## Figures and Tables

**Table 1:** Summary of the published studies (including >1 patient) on positron emission tomography (PET) in Merkel cell carcinoma (in chronological order).

Author (Year)	Study Design	No. of Patients (Mean Age; % Male)	PET Radiotracers	Main Findings
Scanga et al. (2004) [14]	Retrospective	2 (n.r.)	^18^F-FDG	1 TP patient, 1 TN patient
Yao et al. (2005) [15]	Report of 2 cases	2 (68 years; 100%)	^18^F-FDG	Pre-treatment PET scans revealed metastatic disease not detected in CT. Post-treatment PET imaging predicted response to therapy.
Talbot et al. (2005) [16]	Case series	3 (63 years; 67%)	^18^F-FDOPA ^18^F-FDG	2 TP cases with both ^18^F-FDOPA and ^18^F-FDG. No uptake of ^18^F-FDOPA, and unclear findings with ^18^F-FDG in the TN patient. Lower image contrast with ^18^F-FDOPA compared to ^18^F-FDG.
Belhocine et al. (2006) [17]	Retrospective	11 (64 years; 36%)	^18^F-FDG	Sensitivity 92% (11 TP, 1 FN), specificity 100% (3 TN, 0 FP). Contributive PET findings in 10/11 MCC cases.
Iagaru et al. (2006) [18]	Retrospective case series	6 (69 years; 67%)	^18^F-FDG	9 TP, 7 TN, 1 FP, and 1 FN lesions.
Concannon et al. (2009) [19]	Retrospective	18 (74 years; 67%)	^18^F-FDG	PET/CT altered staging in 33% and management in 43% of cases. Sensitivity 94%.
Peloschek et al. (2010) [20]	Retrospective	16 (75 years; 69%)	^18^F-FDG (16 pts.) ^18^F-FDOPA (5 pts)	^18^F-FDG: Sensitivity 85.7%, specificity 96.2% ^18^F-FDOPA: Negative findings in all cases (19 TN, 2 FN).
Maury et al. (2011) [21]	Retrospective	15 (68 years; 60%)	^18^F-FDG	PET/CT had significant impact on staging and management in 46% of cases vs. clinical examination alone. Sensitivity, specificity, PPV, and NPV were 89%, 100%, 100%, and 93%, respectively.
Lu et al. (2012) [22]	Retrospective	9 (70 years; 78%)	^18^F-FDG	^18^F-FDG PET/CT detected more lesions and staged patients more accurately than ^111^In-Pentetreotide scintigraphy. 6 TP, and 4 TN scans.
Colgan et al. (2012) [23]	Retrospective	33 (70 years; 72%) *	^18^F-FDG	Sensitivity 83%, specificity 95%, PPV 91%, NPV 91% in detecting nodal basin disease.
Hawryluk et al. (2012) [24]	Retrospective	97 (70 years; 58%)	^18^F-FDG	PET/CT detected regional nodal disease in 14% of patients, and upstaged 16% of more advanced cases.
Schmidt et al. (2012) [25]	Report of 2 cases	2 (72 years; 50%)	^68^Ga-DOTATATE	Disease extent was determined in both cases leading to management changes with inclusion of DOTATATE-peptide receptor radiotherapy in the therapeutic regimen.
Siva et al. (2013) [26]	Retrospective	102 (77 years; n.r.)	^18^F-FDG	PET-based staging had a significant impact on management in 37% of cases. High- and medium-impact scans were recorded for 22% and 15% of patients, respectively. PET staging results differed from conventional staging results in 22% of patients. In stratification by PET-defined stage, the 5-year OS was 67% in stage I/II patients and 31% in stage III cases (log-rank *p* < 0.001). On multivariate analysis, PET staging was significantly associated with OS (*p* < 0.001).
Ibrahim et al. (2013) [27]	Retrospective	20 (58 years; 45%)	^18^F-FDG	Changes in tumor status and management occurred in 20% and 15% of cases, respectively, as a direct result of PET/CT.
George et al. (2014) [28]	Retrospective	23 (74 years ^#^; 57%)	^18^F-FDG	Sensitivity 97%, specificity 89%, PPV 94%, NPV 94%, with 2 FP and 1 FN results. Lesions neglected clinically or by conventional imaging were revealed in 44% of PET/CTs at initial presentation and during follow-up, with, respectively, 50% and 41% of scans identifying new lesions. At initial presentation, PET/CT altered tumor staging in 39% of cases. Management was modified by PET/CT in one-third of cases (33% at initial presentation; 32% during follow-up; 36% during evaluation of chemotherapy response).
Buder et al. (2014) [29]	Retrospective	24 (68 years; 67%)	^68^Ga-DOTATOC ^68^Ga-DOTATATE	Sensitivity 73% for nodal, 100% for bone, and 67% for soft-tissue metastases. Up-staging and management changes in 17% and 13% of cases, respectively, as a result of PET.
Byrne et al. (2015) [30]	Retrospective	62 (n.r.)	^18^F-FDG	The impact of PET on disease restaging was high in 45% and medium in 11% of cases, respectively. Patients who achieved no CMR had a 15% 1-year OS, while those with CMR had an 88% 2-year and a 68% 5-year OS. Both CMR achievement and nodal disease were significantly prognostic of the OS.
Sollini et al. (2015) [31]	Retrospective	23 (70 years; 78%)	^68^Ga-DOTATOC ^68^Ga-DOTANOC ^68^Ga-DOTATATE	11 TP, 8 TN, 3 FP, and 1 FN cases. Sensitivity 92%, specificity 73%, and diagnostic accuracy 83%. Impact on management in 30% of cases.
Ben-Haim et al. (2016) [32]	Retrospective	46 (68 years; 61%)	^18^F-FDG	PET/CT altered disease stage in 26% resulting in management changes in 15% of cases.
Liu et al. (2017) [33]	Retrospective	16 (69 years; 75%)	^18^F-FDG	In stage I-II MCC, PET/CT was less sensitive (6% positive results) vs. SLNB (63% positive results) in detecting occult nodal metastasis.
Poulsen et al. (2017) [34]	Prospective	58 (68 years; 78%)	^18^F-FDG	Sensitivity 95%, specificity 88%, PPV 95%, NPV 88%. Pre-treatment PET impacted treatment decisions in 27.6% of cases, leading to upstaging in 25.9% of them.
Taralli et al. (2018) [35]	Retrospective	15 (70 years; 80%)	^18^F-FDG ^68^Ga-DOTATOC ^68^Ga-DOTANOC ^68^Ga-DOTATATE	On patient-based analysis, ^18^F-FDG and ^68^Ga-somatostatin analogs showed both 100% sensitivity, and 85.7% and 71.4% specificity, respectively, without significant difference. On lesion-based analysis, ^18^F-FDG detected 89% and ^68^Ga-somatostatin analogs 92% of the lesions, without significant difference.
Singh et al. (2020) [10]	Retrospective	352 (nr.; nr.)	^18^F-FDG	PET/CT upstaged 16.8% of patients. Higher sensitivity vs. CT. Baseline imaging led to upstaging also in patients with clinically uninvolved regional nodes.

TP, true positive; TN, true negative; FP, false positive; FN, false negative; PPV, positive predictive value; NPV, negative predictive value; OS, overall survival; SLNB, sentinel lymph node biopsy; MCC, Merkel cell carcinoma; CMR, complete metabolic response; n.r., not reported. * The numbers refer to the whole study population of 99 patients. ^#^ Median age (mean age not provided).

**Table 2:** Summary of the published studies (including >1 patient) presenting a direct comparison between ^18^F-fluorodeoxyglucose (^18^F-FDG) PET or ^18^F-FDG PET/CT and CT in Merkel cell carcinoma (in chronological order).

Author (Year)	No. of Patients Undergoing PET/No. of Patients Undergoing CT	Main Findings
Yao et al. (2005) [15]	2/2	Pre-treatment PET detected metastatic disease in both patients not appreciated in CT.
Peloschek et al. (2010) [20]	16/16	PET: Sensitivity 85.7%, specificity 96.2% Morphological imaging (CT, MRI, US): Combined sensitivity 95.5%, combined specificity 89.1%.
Maury et al. (2011) [21]	15/15	PET: Sensitivity 89%, specificity 100%, PPV 100%, NPV 93%. CT: Sensitivity 89%, specificity 100%, PPV 100%, NPV 93%.
Colgan et al. (2012) [23]	33/69	PET and PET/CT: Sensitivity 83%, specificity 95%, PPV 91%, NPV 91% in detecting nodal basin disease. CT: Sensitivity 47%, specificity 97%, PPV 94%, NPV 68% in detecting nodal basin disease. PET was significantly more sensitive and equally specific in comparison with CT.
Hawryluk et al. (2012) [24]	97/97	Bone/bone marrow metastases in 10 cases were revealed only on PET with no CT correlate.
George et al. (2014) [28]	23/n.r.	All lesions identified by CT were also detected by PET. Lesions not detected clinically or by conventional imaging (not further specified) were found in 44% of PET/CTs performed at initial presentation and subsequent monitoring with, respectively, 50% and 41% of scans identifying new lesions.
Poulsen et al. (2017) [34]	58/58	PET led to upstaging in 15 (25.9%) of patients, with no cases of downstaging. Upstaging was due to detection of distant metastases (4 cases) or regional nodes (6 cases) that were not reported on CT.
Singh et al. (2020) [10]	352/231	PET/CT upstaged patients (16.8% of 352) significantly more often than CT alone (6.9% of 231).

US, ultrasound; PPV, positive predictive value; NPV, negative predictive value; n.r., not reported.
